# Low hydrostatic pressure promotes functional homeostasis of nucleus pulposus cells through the TRPV4/CRT/FA complex axis

**DOI:** 10.3389/fmed.2025.1531907

**Published:** 2025-01-29

**Authors:** Junxian Hu, Yibo Zhu, Zeyu Pang, Xiaoxiao Li, Huilin Zhang, Xiangwei Li, Yongjian Gao, Yiyang Wang, Pei Li, Qiang Zhou

**Affiliations:** ^1^Department of Orthopedics, The Third Affiliated Hospital of Chongqing Medical University, Chongqing, China; ^2^Tissue Repairing and Biotechnology Research Center, The Third Affiliated Hospital of Chongqing Medical University, Chongqing, China

**Keywords:** intervertebral discs, nucleus pulposus cell, hydrostatic pressure, calreticulin, cellular functional homeostasis

## Abstract

The low hydrostatic pressure in the intervertebral disc plays a crucial role in maintaining the homeostasis of the disc environment, particularly in supporting the physiological functions of nucleus pulposus cells (NPCs). However, the underlying mechanisms remain poorly understood. TRPV4, a baroreceptor in the intervertebral disc, is primarily responsible for converting extracellular pressure signals into intracellular chemical signals. Upon activation, TRPV4 facilitates the influx of calcium ions, thereby regulating the physiological behavior of NP cells. Calreticulin (CRT), an endoplasmic reticulum retention protein, performs various physiological functions, including the regulation of intracellular calcium levels. CRT also exhibits distinct roles depending on its subcellular localization. In this study, we observed that under low hydrostatic pressure, TRPV4 activation and subsequent calcium influx led to an increase in CRT synthesis and a significant rise in its cytosolic expression. This was followed by the depolymerization of focal adhesion (FA) complexes, primarily consisting of FAK and integrin β1, which resulted in an increase in collagen type II (Col II) and a decrease in collagen type I (Col I). These changes in extracellular matrix (ECM) composition helped maintain the physiological function of NP cells. Furthermore, overexpression of CRT enhanced the ability of NP cells to resist partial functional damage caused by high hydrostatic pressure. Taken together, our findings suggested that low hydrostatic pressure enhanced NP cell function by regulating the TRPV4/CRT/FA complex signaling axis.

## 1 Introduction

The homeostasis of the intervertebral disc environment is particularly important for the maintenance of its function (1). The intervertebral disc comprises the outer annulus fibrosus (AF), the upper and lower endplates, and the central nucleus pulposus (NP) (2). Among these components, the gelatinous nucleus pulposus, located at the center of the disc, is typically the first to exhibit degenerative changes (3). The unique biomechanical properties of the nucleus pulposus tissue are attributed to the molecular composition and structural characteristics of its functional ECM, which is primarily secreted by nucleus pulposus cells (4). The functional ECM is largely composed of a network of collagen fibers (predominantly type II collagen, (Col II) interwoven with proteoglycans (PGs) and glycosaminoglycans (GAGs). Col II forms a rigid scaffold that supports cell growth, while the hydrophilic PGs and GAGs confer viscoelasticity to the tissue by binding water. This dual-network structure enables the nucleus pulposus to effectively buffer mechanical loads from the spine (5). Some typical pathological features of intervertebral disc degeneration include increased apoptosis of nucleus pulposus cells, alterations in the extracellular matrix (ECM), and an upregulation of catabolic processes. As degeneration of the nucleus pulposus progresses, the disc gradually loses its mechanical cushioning function, ultimately contributing to intervertebral disc degeneration diseases (6).

The intervertebral disc experiences varying levels of hydrostatic pressure daily. Hydrostatic pressure plays a crucial role in both the development and degeneration of the disc intervertebral (7). Mechanical loading on intervertebral disc induces changes in the mechanical pressure (tissue pressure) and hydrostatic pressure (fluid pressure) within the ECM, which, in turn, influences the biological behavior of nucleus pulposus cells (8). Previous studies have demonstrated that high hydrostatic pressure accelerates nucleus pulposus cell senescence and apoptosis, thus promoting disc degeneration (9). Additionally, this stress induces changes in the intervertebral disc’s matrix, particularly in the collagen content. Type I collagen (Col I), which predominates in the annulus fibrosus, increases in the nucleus pulposus during early degeneration, while Col II, typically found in the inner nucleus pulposus, decreases (10). Col I is involved in tissue repair and fibrosis, contributing to the hyperplasia of connective tissue and the ossification of the nucleus pulposus. However, as degeneration progresses, the activity of matrix metalloproteinases (MMPs) increases, leading to the degradation of Col I. This degradation further diminishes the disc’s resistance to mechanical stress, thus exacerbating the degenerative process. In addition to causing disc degeneration, many studies also suggest that appropriate low hydrostatic pressure within intervertebral disc not only contribute to the development of the intervertebral disc but also play a crucial role in maintaining the internal environment and functional homeostasis of the discs (11, 12). Consistent with previous literature, our research group has shown that when hydrostatic pressure was below 0.5 MPa, the secretion of Col II by the NPCs in the inner layer of the intervertebral disc was significantly enhanced. However, when the pressure exceeded 1.0 MPa, there was a notable decrease in Col II production, accompanied by an increase in Col I, suggesting that the optimal pressure for promoting NPCs activity is under 0.5 MPa. Beyond 1.0 MPa, the pressure experienced by NP cells can be classified as high loading pressure, which accelerated intervertebral disc degeneration. Pressures between 0.5 and 1.0 MPa represented an intermediate range that did not significantly alter the physiological behavior of NP cells. However, the precise mechanisms by how hydrostatic pressures below 0.5 MPa promoted NP cell function remain poorly understood and require further investigation (13).

Calreticulin (CRT) is a key endoplasmic reticulum (ER) retention protein, but it is also found in the cytoplasm, cell membrane, and extracellular space (14). The subcellular localization of calreticulin influences its physiological functions. For instance, in the ER, calreticulin promotes cellular homeostasis by facilitating correct protein folding (15). On the cell membrane, extracellular calreticulin induces apoptosis and activates immune pathways, while in the extracellular space, it plays a role in skin repair, among other functions (16). An additional critical role of calreticulin is its ability to bind calcium ions, regulate intracellular calcium concentrations, and sense fluctuations in calcium levels. The conformation of calcium-bound calreticulin undergoes changes that enable it to perform specific functions (17).

The transient receptor potential vanilloid 4 (TRPV4) is a mechanosensitive receptor in the cell membrane that converts extracellular pressure signals into intracellular physiological responses. When nucleus pulposus cells experience stress, the TRPV4 channel opens, allowing calcium influx (18). While controlled calcium influx is necessary for normal cellular function, excessive calcium entry can lead to irreversible cell damage. Therefore, variations in intracellular calcium flux, driven by low or high pressure, likely regulate the behavior of different nucleus pulposus cells (19). We hypothesized that under low stress, TRPV4 opening facilitated proper calcium influx, enabling calreticulin to function as a biological promoter. However, excessive calcium influx may overwhelm calreticulin’s capacity to regulate calcium, ultimately leading to cellular dysfunction.

This study investigated the biological effects of low hydrostatic pressure on calcium influx and calreticulin. The results suggested that low hydrostatic pressure, TRPV4 channel activation mediated appropriate calcium influx, which promoted calreticulin synthesis and its translocation from the ER to the cytoplasm. This process mitigated extracellular matrix fibrosis by inhibiting focal adhesion (FA) complex formation finally.

## 2 Materials and methods

### 2.1 Nucleus pulposus cell culture

Nucleus pulposus cells were isolated from 8-week-old Sprague-Dawley (SD) rats. Initially, the soft tissue surrounding the tail was carefully dissected to expose the intervertebral disc. The nucleus pulposus tissue was excised by transecting the intervertebral disc, then minced using ophthalmic scissors and digested with 0.2% collagenase at 37°C for 30 min. After digestion, the resulting supernatant was removed by centrifugation, and the cell pellet was resuspended in complete medium. The cells were then cultured in a humidified incubator at 37°C with 5% CO2. Cells were passaged upon reaching 90% confluence, and passages 2–3 were used for subsequent experiments. This procedure was approved by the Institutional Animal Care and Use Committee (IACUC) of Chongqing Medical University (CQMU).

### 2.2 Hydrostatic culture of cells

Following trypsin digestion, cells were resuspended and centrifuged before being seeded onto a custom-made chamber with a polycarbonate membrane. The chamber was placed into a self-designed bioreactor filled with complete medium. Hydrostatic pressure was applied longitudinally using a force applicator, with a pressure gauge connected at one end of the reactor to monitor the pressure. Hydrostatic pressure was applied for 5 h daily, with the reactor opened every 2 h to ensure adequate oxygenation.

### 2.3 Calcium ion detection

Samples were washed three times with phosphate-buffered saline (PBS), then incubated with calcium ion detection kit (Beyotime, China) according to the manufacturer’s instructions at 37°C for 30 min. After incubation, the samples were fixed with 4% paraformaldehyde for 5 minutes and subsequently mounted with DAPI- or Hoechst-containing mounting solution (Beyotime, China). Fluorescence expression was observed using fluorescence or confocal microscopy.

### 2.4 Immunofluorescence staining

Treated cells were fixed with 4% paraformaldehyde (Biosharp, China) for 15 minutes and washed three times with PBS (BIOFIL, China). The cell membranes were permeabilized using Triton X-100 for 3 min (Beyotime, China), followed by blocking with a rapid immunofluorescence blocking solution (Beyotime, China) for 15 min. Primary antibodies (Proteintech, China), prepared in advance, were added to the samples, which were then incubated at 4°C overnight. The following day, the samples were washed three times with PBS and incubated with fluorescent-conjugated secondary antibodies (Proteintech, China) at 37°C for 1 h. After washing three times with PBS, the samples were mounted with a DAPI- or Hoechst-containing mounting solution and examined under fluorescence or confocal microscopy.

### 2.5 Cytoplasmic protein extraction

The cell supernatant was removed, and components other than the cytoplasm (including the cell membrane) were isolated using the Minute™ Plasma Membrane Protein and Cell Fractionation Kit (Invent, China), following the manufacturer’s instructions. After the addition of loading buffer, the sample was heated at 100°C for 10 min and then stored at −20°C for future use.

### 2.6 Protein extraction and Western blotting

Western blotting was performed as follows: Total protein was extracted using a protein lysis buffer containing 1% PMSF. Proteins were separated by SDS-PAGE and transferred to PVDF membranes (Millipore, United States). The membranes were then blocked with an antibody blocking solution for 1 h, followed by incubation with primary antibodies diluted in blocking solution overnight at 4°C. The primary antibodies (Proteintech, China) used were as follows: Col2 (1:1,000), Col1 (1:1,000), ACAN (1:1,000), FAK (1:1,000), ITGβ1 (1:1,000), β-actin (1:5,000), CRT (1:1,000), and TRPV4 (1:1,000). After primary antibody incubation, the membranes were incubated with a secondary antibody (Proteintech, China) for 1 h at room temperature and then washed three times with TBST for 15 min each. Protein expression was detected using an appropriate imaging system.

### 2.7 Cell transfection

Nucleus pulposus (NP) cells were seeded into 6-well plates and cultured until they reached 50–55% confluence. The cells were then transfected with recombinant lentiviral vectors (LV-CRT, Genechem, China) for 12 h to induce CRT expression. Control NP cells were transfected with negative control vectors (LV-NC). Transfection efficiency was assessed by fluorescence microscopy and Western blot analysis.

### 2.8 RT-PCR

Total RNA was extracted from NP cells using TRIZOL reagent (Invitrogen, United States), according to the manufacturer’s instructions. RNA concentration was determined, and the extracted RNA was reverse transcribed into complementary DNA (cDNA) using a reverse transcription kit (Roche, Switzerland). Quantitative real-time PCR (qRT-PCR) was performed to quantify mRNA expression. The PCR reaction mixture included cDNA, specific primers (listed in Table 1), and SYBR Green Master Mix (Roche, Switzerland), with amplification carried out for 40 cycles. β-Actin was used as the internal reference gene, and relative gene expression levels were calculated using the 2-ΔΔCt method.

**TABLE 1:** Primers of target genes.

Gene	Forward (5′–3′)	Reverse (5′–3′)
GAPDH	GCAAGTTCAACGG CACAG	CGCCAGTAGAC TCCACGAC
MMP13	CAAGATGTGGAGTGCC TGATGTGG	GCGTGTGCCAGAAG ACCAGAAG
ACAN	GCGTGTGCCAGAAG ACCAGAAG	ATGTCCTCTTCACCA CCCACTCC
COL II	GGAGCAGCAAGAG CAAGGAGAAG	GGAGCCCTCAGTG GACAGTAGAC

### 2.9 Statistical analysis

All experiments were conducted with at least three replicates. Statistical analysis was performed using SPSS version 20.0, and data are expressed as mean ± standard deviation (SD) (*n* ≥ 3). Comparisons between groups were made using t-tests or one-way analysis of variance (ANOVA). A *p*-value of < 0.05 was considered statistically significant.

### 2.10 Mechanism diagram

This figure was created by Figdraw.^1^

## 3 Results

### 3.1 Low hydrostatic pressure regulated extracellular matrix fibrosis in nucleus pulposus cells

**FIGURE 1:**
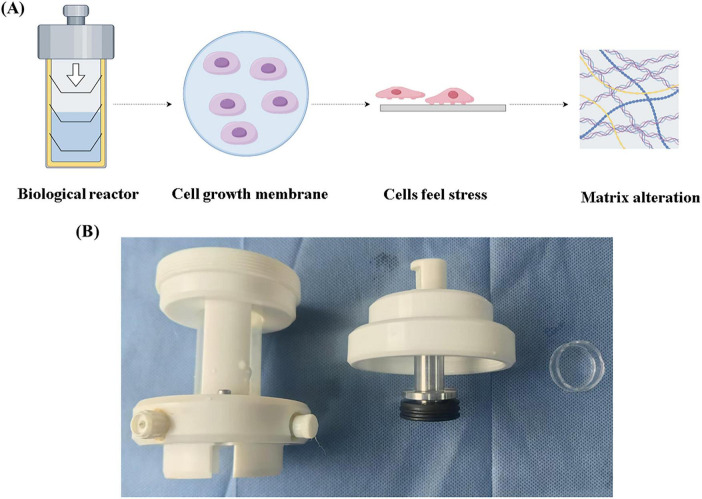
Self-developed bioreactor. **(A)** Schematic diagram of the bioreactor. **(B)** Bioreactor actual device.

**FIGURE 2:**
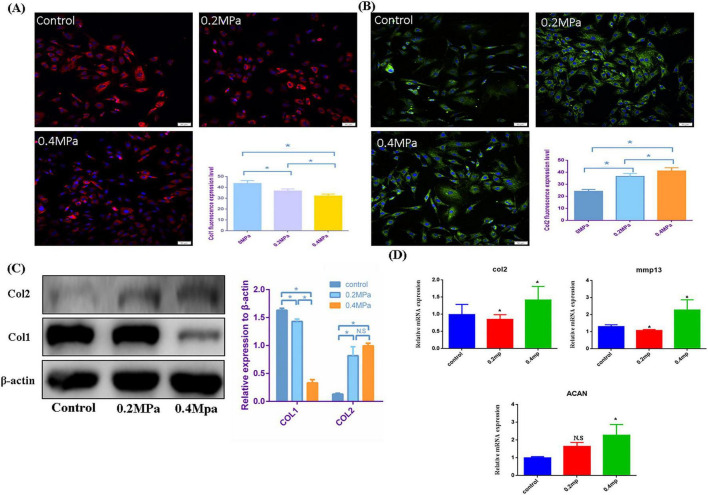
Effect of low pressure on the secretory function of nucleus pulposus cells. **(A)** Immunofluorescence analysis and quantitative assessment of COL I expression. **(B)** Immunofluorescence analysis and quantitative assessment of COL II expression. **(C)** Western blot analysis showing changes in COL II, and COL I secretion following different pressure treatments, along with corresponding quantitative data. **(D)** Quantitative PCR (qPCR) analysis of MMP13, COL II, and ACAN gene expression in response to various pressure stimuli. *p < 0.05.

To investigate the effect of low hydrostatic pressure on matrix secretion in nucleus pulposus cells, we developed a hydrostatic pressure system that applied varying levels of pressure to cells cultured on a biofilm. The schematic and physical models of the hydrostatic device are shown in Figures 1A,B. First, we applied physiological low-level hydrostatic pressure (ranging in strength) to the cells and performed immunofluorescence staining after five days of exposure. The results indicated that at 0.2 and 0.4 MPa, the expression of COL I decreased as pressure increased, while the expression of COL II increased with pressure (see Figures 2A,B). Next, we subjected the cells to the same pressure conditions and extracted proteins for Western blot analysis (Figure 2C). The findings corroborated the immunofluorescence data: COL II levels significantly increased under hydrostatic pressure, while COL I levels decreased. qPCR analysis revealed that under low hydrostatic pressure, both ACAN and Col2 levels increased, while MMP13 expression decreased at 0.2 MPa and slightly increased at 0.4 MPa (Figure 2D). These results suggested that physiological low hydrostatic pressure enhanced the secretion of COL II by nucleus pulposus cells, reduced the secretion of COL I, and mitigated extracellular matrix fibrosis.

### 3.2 Low pressure stress alleviated extracellular matrix fibrosis induced by high pressure

**FIGURE 3:**
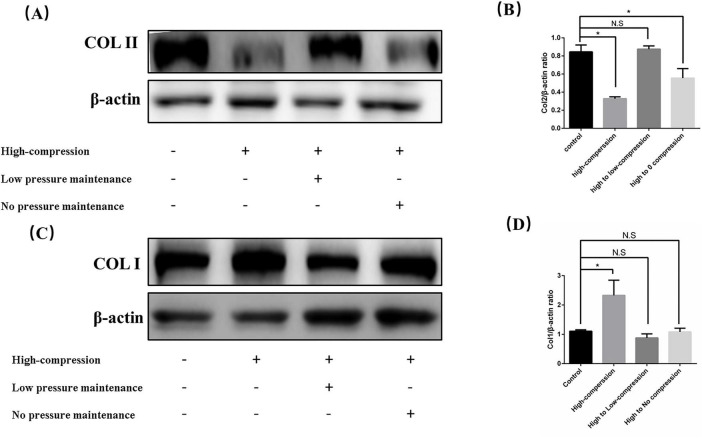
Low hydrostatic pressure mitigated functional damage to nucleus pulposus cells induced by high pressure. **(A)** Western blot analysis showing the effects of various stress unloading methods on the synthesis of COL II following high hydrostatic pressure treatment. **(B)** Quantitative analysis of Western blot results. **(C)** Western blot analysis showing the effects of various stress unloading methods on the synthesis of COL I following high hydrostatic pressure treatment. **(D)** Quantitative analysis of Western blot results. *p < 0.05.

To further explore whether low pressure can counteract extracellular matrix fibrosis induced by high pressure, we established a high-pressure unloading model. The experiment consisted of four groups: the control group (no treatment), the high-pressure group (constant high-pressure application), the high to low-pressure group (high pressure was replaced by low pressure after 2 days), and the high to no-pressure group (high pressure was replaced by 0 MPa after 2 days). After five days of sample collection, Western blot analysis (Figures 3A–D) revealed that COL II levels were significantly decreased, while COL I increased in the continuous high-pressure group. These findings are consistent with prior reports, indicating that prolonged high-pressure exposure leads to extracellular matrix fibrosis in nucleus pulposus cells as a protective response to the stress. Upon comparison of the high to low-pressure group and the high to no-pressure group, we observed that COL II levels recovered in the unloading groups, while COL I decreased. Notably, the degree of fibrosis in the extracellular matrix was less pronounced in the high to low-pressure group than in the high to no-pressure group. Overall, these results demonstrated that maintaining low pressure can alleviate the extracellular matrix fibrosis induced by high pressure.

### 3.3 Low pressure stimulated calreticulin synthesis via TRPV4 pathway activation and calcium influx

**FIGURE 4:**
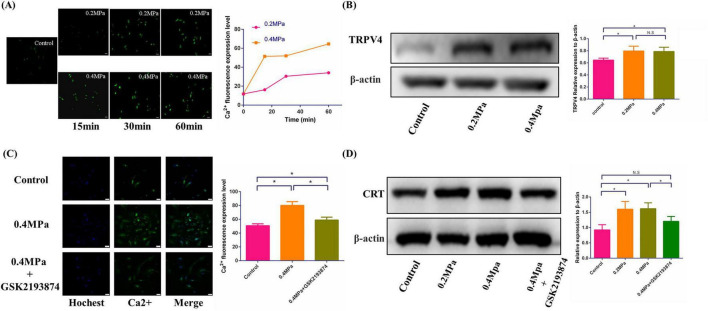
Low hydrostatic pressure induced calcium influx and enhances calreticulin synthesis through upregulation of TRPV4 expression. **(A)** Temporal analysis of calcium influx under low hydrostatic pressure, along with statistical evaluation (scale bar = 50 μm). **(B)** TRPV4 protein expression under low hydrostatic pressure and corresponding statistical analysis. **(C)** Calcium influx and statistical analysis following administration of a TRPV4 inhibitor. **(D)** Low hydrostatic pressure promoted calreticulin synthesis, with statistical analysis. *p < 0.05.

Cells typically transduce mechanical pressure signals into intracellular responses through the activation of pressure-sensitive receptors on the cell membrane. This process triggers calcium ion influx, which in turn influences various cellular physiological and pathological processes. To investigate the effect of different pressure levels on calcium influx, we subjected cells to 15, 30, and 60 min of pressure application and measured calcium influx at each time point (Figure 4A). Our results indicated that calcium influx increased over time, with a significantly higher influx at 0.4 MPa compared to 0.2 MPa. Next, we examined the expression of the pressure-sensitive receptor TRPV4 under varying pressure conditions. We found that TRPV4 expression was markedly elevated under pressure, suggesting that hydrostatic pressure may activate TRPV4, promoting calcium influx to regulate the physiological state of nucleus pulposus cells (Figure 4B). To further confirm this relationship, we inhibited TRPV4 expression using GSK2193874, a specific TRPV4 inhibitor, and reassessed calcium influx. The results showed a significant reduction in calcium influx at 0.4 MPa in the inhibitor-treated group compared to the 0.4 MPa group, indicating that TRPV4 activation plays a key role in the pressure-induced calcium influx (Figure 4C). Calreticulin, a calcium-binding protein, senses fluctuations in intracellular calcium levels and helps maintain calcium homeostasis. We observed a significant upregulation of calreticulin expression at both 0.2 and 0.4 MPa, and this upregulation was inhibited by the TRPV4 inhibitor (Figure 4D). These findings suggested that hydrostatic pressure increased intracellular calcium levels by upregulating TRPV4 expression, which in turn drives the synthesis of calreticulin to regulate calcium ion concentrations.

### 3.4 Increased calreticulin expression under low pressure inhibited FA complex formation

**FIGURE 5:**
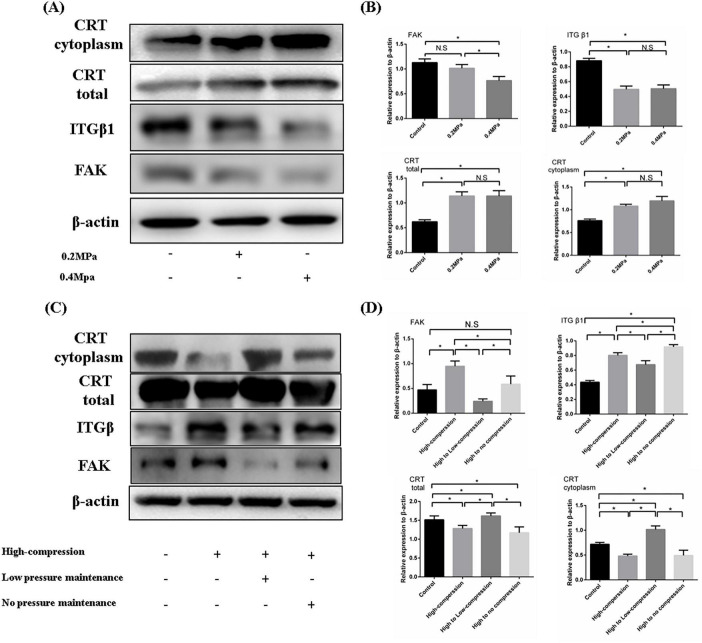
**(A)** Western blotting was employed to assess the composition and expression levels of FA complexes under varying low pressure conditions. **(B)** Quantitative analysis of the WB results. **(C)** WB was used to evaluate the impact of different unloading methods on FA complex formation following high-pressure-induced degeneration of nucleus pulposus cells. **(D)** Quantitative analysis of the WB results. *p < 0.05.

Calreticulin performs diverse functions in different subcellular compartments, such as assisting protein folding in the endoplasmic reticulum and mediating macrophage recognition at the cell membrane. Additionally, calreticulin has functional roles in the cytoplasm. We found that both total calreticulin levels and its cytoplasmic concentration increased significantly under 0.2 and 0.4 MPa pressure conditions. Concurrently, the expression of focal adhesion (FA) complex components, including ITGβ1, and FAK, was reduced (Figures 5A,B), suggesting that increased calreticulin in the cytoplasm promotes FA complex depolymerization. Further investigation of calreticulin and FA complex expression in cells subjected to high-pressure (1 MPa) unloading and a high-to-low pressure transition revealed interesting trends. Although low pressure enhanced calreticulin synthesis, high pressure led to a marked reduction in its expression. Interestingly, calreticulin levels were higher in the high to low pressure group compared to the high to no pressure group. Furthermore, the FA complex expression in the high to low pressure group was significantly lower than in the other high-pressure groups (Figures 5C,D). These results demonstrated that calreticulin accumulated and FA complex formation was inhibited under low-pressure conditions. However, high pressure reduces calreticulin expression, and transitioning from high to low pressure helped maintain calreticulin levels while further decreasing FA complex formation. These findings underscored the dynamic regulation of calreticulin in response to hydrostatic pressure and its role in modulating cell behavior through FA complex.

### 3.5 Overexpression of calreticulin prevented extracellular matrix fibrosis induced by high hydrostatic pressure

**FIGURE 6:**
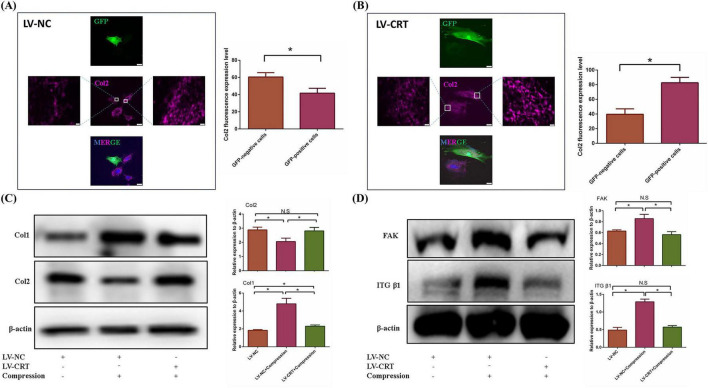
Overexpression of CRT prevented the dysfunction of nucleus pulposus cells induced by high pressure. **(A)** Immunofluorescence of Col2 changed between nucleus pulposus cells transfected with negative lentivirus and non-transfected cells. **(B)** Immunofluorescence of COl2 changed between nucleus pulposus cells transfected with LV-CRT and non-transfected cells. **(C)** The resistance of nucleus pulposus cells to high pressure induced fibrosis of nucleus pulposus extracellular matrix was detected by WB and quantitative analysis. **(D)** WB was used to examine the resistance of CRT overexpression to elevated FA complexes induced by high pressure and quantitative analysis. *p < 0.05.

To investigate the effects of calreticulin (CRT) overexpression on NPCs, we first confirmed successful transfection of CRT in nucleus pulposus (NP) cells using fluorescence microscopy and Western blot analysis (Supplementary Figure S1). COL II staining revealed that NP cells transfected with the LV-NC exhibited significantly lower levels of type II collagen compared to non-transfected cells in the surrounding areas, indicating that lentiviral transfection impacted the state of the NP cells (Figure 6A). In contrast, NP cells overexpressing CRT showed a marked increase in type II collagen expression compared to non-transfected cells (Figure 6B), confirming that CRT overexpression enhanced the production of functional collagen II. Subsequently, we subjected the transfected NP cells to high hydrostatic pressure and observed that the overexpression of CRT resulted in the depolymerization of extracellular matrix fibers, suggesting a protective role in preventing ECM fibrosis under high-stress conditions (Figure 6C). Finally, CRT overexpression effectively mitigated the accumulation of focal adhesion (FA) complex typically induced by high stress (Figure 6D). The above evidence suggested that nucleus pulposus cells overexpressing CRT can resist the dysfunction caused by high pressure through inhibiting FA complex formation

## 4 Discussion

Hydrostatic pressure plays a crucial role in the development, homeostasis, and degeneration of the intervertebral disc. During the developmental stage, appropriate pressure is essential for the differentiation of notochord cells into mature nucleus pulposus cells (20). Numerous studies have demonstrated that low-level hydrostatic pressure enhances the ability of nucleus pulposus cells to secrete functional extracellular matrix (ECM). However, excessive stress in the intervertebral disc can lead to detrimental effects, including water loss, fibrosis, and even ossification of the disc (21). These adverse outcomes are associated with the senescence and apoptosis of nucleus pulposus cells, as well as ECM fibrosis (22). Despite extensive research, the precise mechanisms by how hydrostatic pressure regulates the homeostasis of nucleus pulposus cells, including its bidirectional effects, remain poorly understood. Identifying key targets involved in hydrostatic pressure regulation of nucleus pulposus cells may offer valuable insights for future tissue engineering strategies aimed at repairing or regenerating the nucleus pulposus.

The external pressure experienced by nucleus pulposus cells is typically transduced into intracellular chemical signals via mechanoreceptors on the cell membrane. TRPV4, a member of the transient receptor potential (TRP) channel family, is a prominent mechanoreceptor involved in this process. Upon activation, TRPV4 channels allow a significant influx of calcium ions, which is essential for regulating various cellular functions (23). While moderate calcium influx is critical for maintaining normal cellular behavior, excessive calcium entry can induce detrimental effects, such as endoplasmic reticulum stress and increased intracellular osmotic pressure, ultimately leading to cell senescence and apoptosis. Thus, the regulation of calcium influx may play a pivotal role in the bidirectional response of nucleus pulposus cells to mechanical pressure. In our experiments, we observed that under 0.2 and 0.4 MPa hydrostatic pressure, TRPV4 channels opened, resulting in an increased calcium influx in nucleus pulposus cells. This was accompanied by a marked decrease in Col I expression and an increase Col II expression. The calcium influx was significantly inhibited when a TRPV4 ion channel inhibitor was applied. Collectively, these findings suggested that while controlled calcium influx is necessary for maintaining nucleus pulposus cell homeostasis.

Calreticulin is an ER retention protein that plays a crucial role in regulating ER function and maintaining cellular homeostasis (24). Depending on its subcellular localization, calreticulin can be secreted and perform distinct functions in various cellular compartments. For instance, it can be translocated from the ER to cytosol or other organelles as needed (25). Calreticulin is also a calcium-binding protein, with multiple calcium ion binding sites. Each calreticulin molecule can bind up to four calcium ions. When intracellular Ca^2 +^ concentrations rise, calreticulin binds to these ions, thereby modulating calcium homeostasis. Conversely, when Ca^2 +^ levels decrease, calreticulin releases calcium ions, returning to its inactive conformation (26). In our study, we observed a significant increase in calreticulin expression in response to low hydrostatic pressure. This suggested that the synthesis of calreticulin is upregulated to regulate intracellular calcium levels under this condition. This increase in calreticulin expression can be inhibited by TRPV4 inhibitors, supporting the hypothesis that TRPV4 indirectly influences calreticulin expression by mediating calcium ion flux. Calreticulin contains an ER retention peptide, a short tail sequence that targets it to the ER. When misfolded proteins or those destined for other organelles contain this peptide, they are redirected back to the ER for disassembly or to perform ER-specific functions. Due to the presence of the ER retention peptide, calreticulin is primarily retained within the ER and is generally not secreted into the cytoplasm or extracellular space unless stimulated (27). Our findings also revealed a significant increase in cytosolic calreticulin in response to low hydrostatic pressure. This increase may be due to an accumulation of cytosolic calcium ions, prompting calreticulin to regulate calcium ion concentrations by facilitating the transfer of calcium from the ER to the cytosol. Interestingly, when we further exposed nucleus pulposus cells to high hydrostatic pressure (1.0 MPa), the intracellular calreticulin content decreased. We hypothesized that, in response to the overwhelming accumulation of calcium ions, calreticulin bind to the excess ions and transported them out of the cell, thereby reducing intracellular calcium concentrations and preventing cellular damage.

Additionally, we observed that after high-pressure-induced dysfunction of nucleus pulposus cells, recovery of calreticulin in these cells under low hydrostatic pressure occurred significantly faster than in cells transitioning from high pressure to no pressure. Low hydrostatic pressure effectively reduced the formation of the focal adhesion (FA) complex by increasing calreticulin synthesis. The FA complex, composed of proteins such as Focal Adhesion Kinase (FAK), integrins, vinculin, and paxillin, plays a key role in regulating cell-extracellular matrix (ECM) interactions. Integrins, in particular, sense changes in the external environment to regulate cell adhesion and various signaling pathways. Previous studies have shown that activation of the FA complex leads to increased expression of TGF-β1, which, at appropriate levels, supports the survival and function of nucleus pulposus cells. However, excessive TGF-β1 expression is associated with accelerated intervertebral disc degeneration and ECM fibrosis (28, 29). Our previous research also demonstrated that FA complex levels were significantly lower in NPCs spheres compared to isolated nucleus pulposus cells, leading to increased COL II synthesis and reduced COL I synthesis (30). In the current experiment, we found that the increase in calreticulin under low pressure was accompanied by a reduction in FA complex formation and a decrease in ECM fibrosis. Conversely, high hydrostatic pressure led to a decrease in calreticulin levels, followed by an increase in FA complex formation. Moreover, overexpression of calreticulin in nucleus pulposus cells resulted in a marked increase in Col II expression compared to non-transfected controls. These calreticulin-overexpressing cells also exhibited greater resistance to the FA complex-induced dysfunction and ECM fibrosis caused by high hydrostatic pressure. These findings suggested that calreticulin plays a protective role by modulating the FA complex and mitigating ECM fibrosis induced by high hydrostatic pressure. In summary, our data suggested that excessive pressure depleted calreticulin, which may contribute to nucleus pulposus cell dysfunction. In contrast, low-pressure conditions promoted calreticulin synthesis, helping to maintain intracellular homeostasis. Thus, calreticulin appears to be a critical regulator of nucleus pulposus cell function and a potential target for therapeutic strategies aimed at mitigating disc degeneration.

**FIGURE 7:**
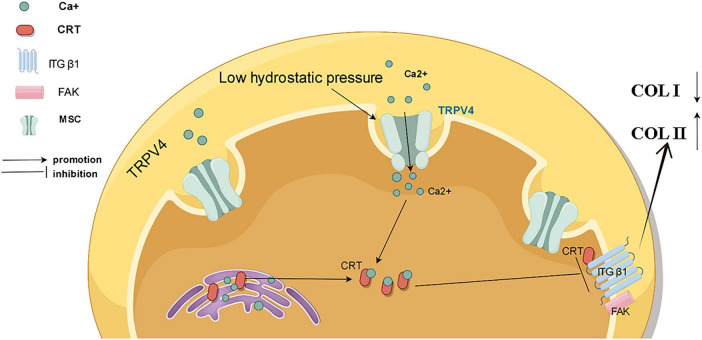
Schematic representation of the mechanism by which low pressure maintains functional homeostasis in nucleus pulposus cells by regulating TRPV4/CRT/FA complexes.

The mechanism by how low pressure regulates the homeostasis of the intervertebral disc environment remains poorly understood. In this study, we demonstrated that low hydrostatic pressure can effectively maintain the functional homeostasis of nucleus pulposus cells through the TRPV4/CRT/FA complex axis (Figure 7). Furthermore, overexpression of calreticulin enhanced the functionality of nucleus pulposus cells, rendering them more resistant to high pressure. These findings offer new insights into the relationship between low hydrostatic pressure and intervertebral disc function and propose a potential protein target for future tissue engineering applications.

## 5 Conclusion

In this experiment, we observed that high hydrostatic pressure exacerbated extracellular matrix fibrosis, while appropriately low hydrostatic pressure was able to inhibit this process. Low hydrostatic pressure regulated the functional homeostasis of nucleus pulposus cells through the TRPV4/CRT/FA complex axis. Furthermore, the study highlighted the crucial role of calreticulin in maintaining the physiological stability of nucleus pulposus cells and their ability to withstand high hydrostatic pressure. Based on these results, future tissue engineering could utilize CRT as a core cytokine for effective repair of degenerative nucleus pulposus.

## Data Availability

The raw data supporting the conclusions of this article will be made available by the authors, without undue reservation.

## Footnotes

1
https://www.figdraw.com/

## Supplementary material

Supplementary Figure S1 CRT transfection. **(A)** The transfection efficiency was observed by immunofluorescence. **(B)** Western blot was used to detect protein expression after CRT transfection.
