# Supplementary figures and images for: Low hydrostatic pressure promotes functional homeostasis of nucleus pulposus cells through the TRPV4/CRT/FA complex axis
Source: Front Med (Lausanne). 2025 Jan 29;12:1531907. doi: 10.3389/fmed.2025.1531907 (PMC11813897; doi:10.3389/fmed.2025.1531907)

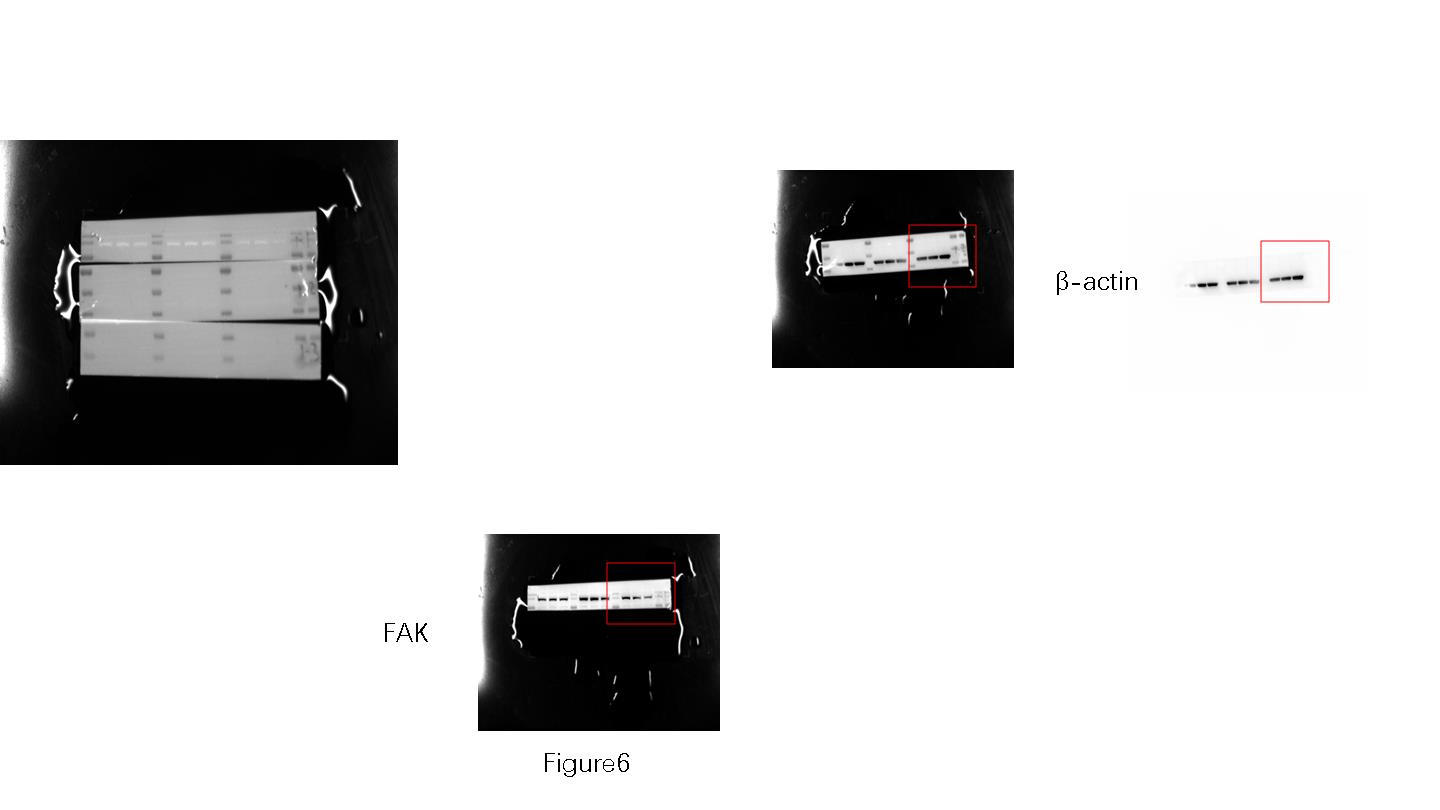

Supplement: Supplementary file 1 [file Data_Sheet_1.zip › 幻灯片16.jpeg]

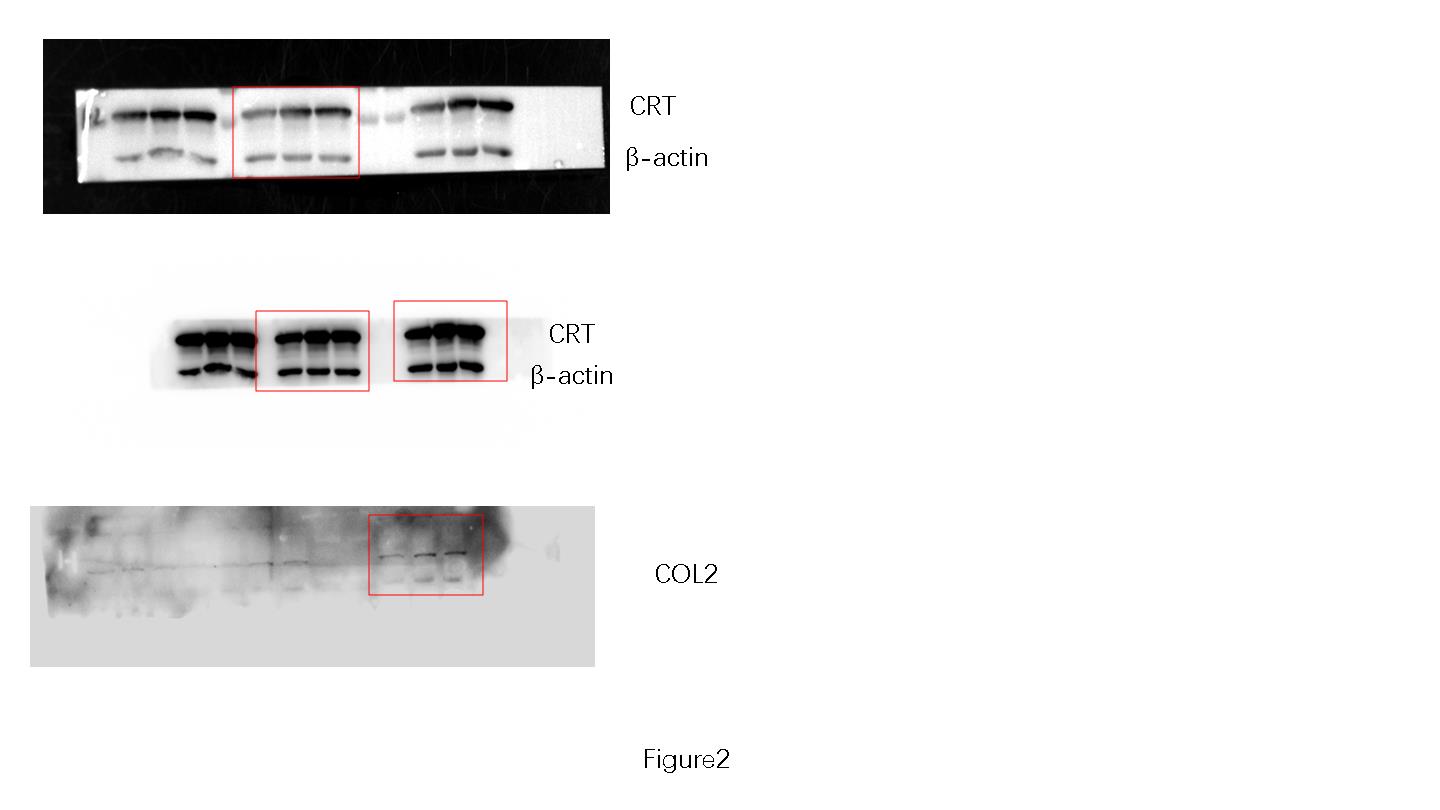

Supplement: Supplementary file 1 [file Data_Sheet_1.zip › 幻灯片1.jpeg]

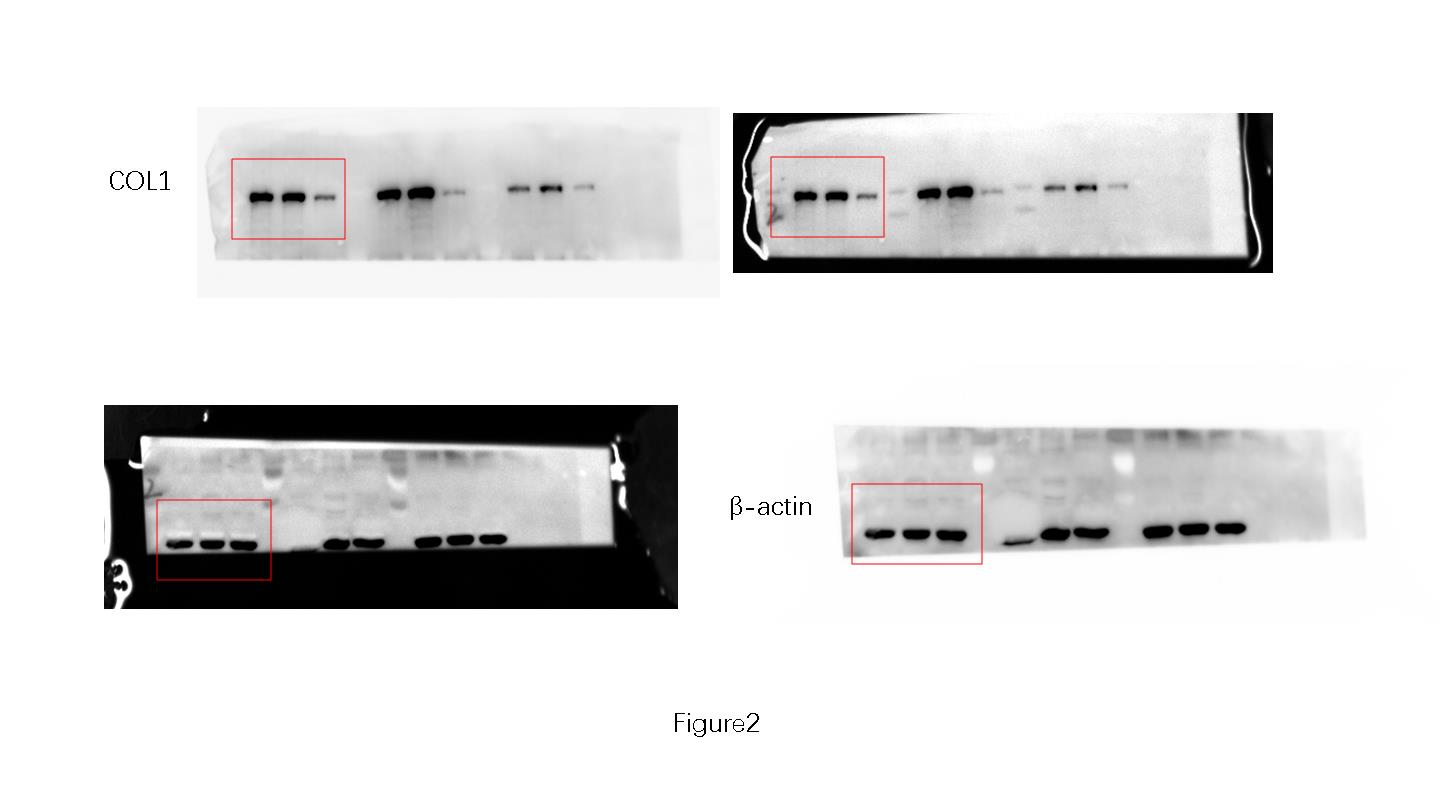

Supplement: Supplementary file 1 [file Data_Sheet_1.zip › 幻灯片2.jpeg]

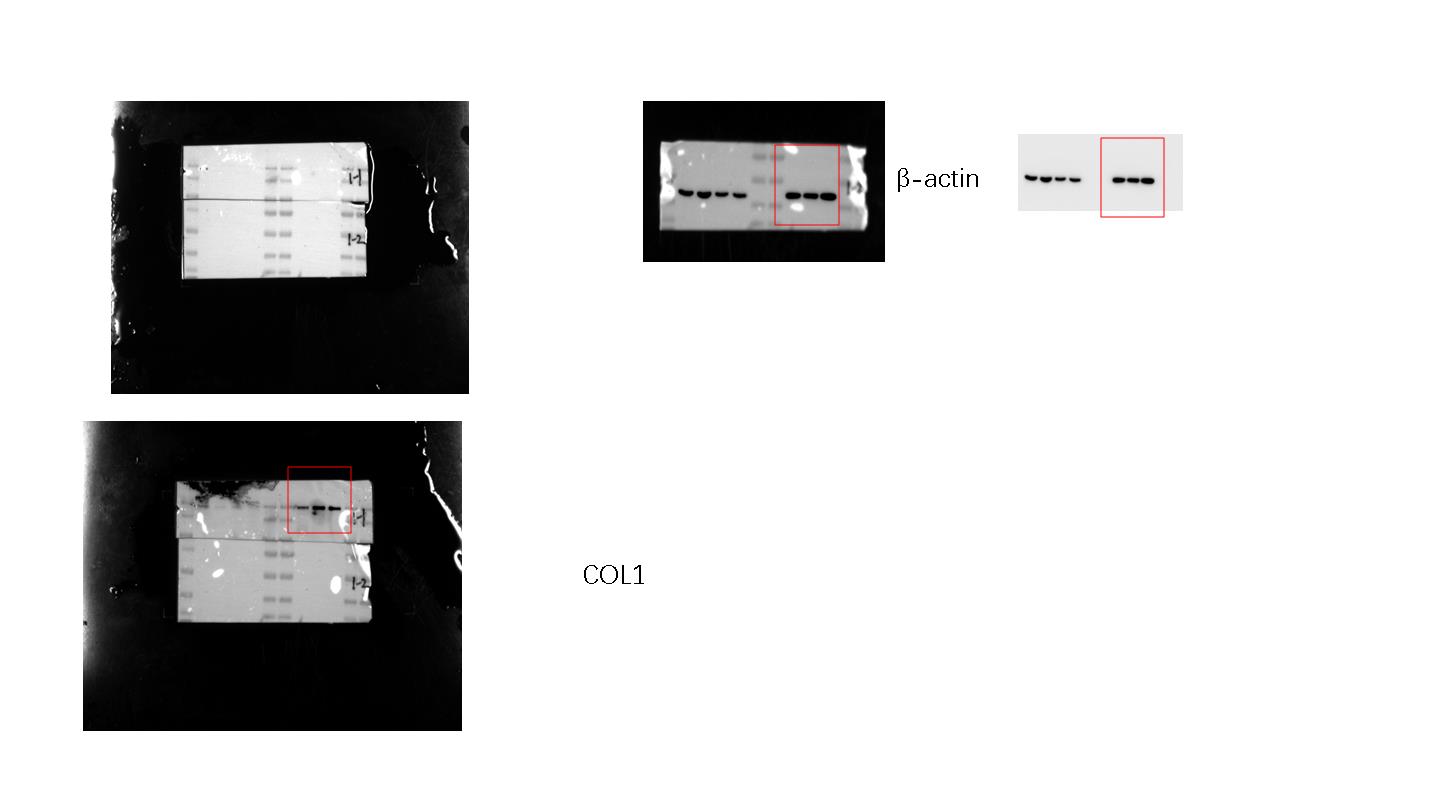

Supplement: Supplementary file 1 [file Data_Sheet_1.zip › 幻灯片3.jpeg]

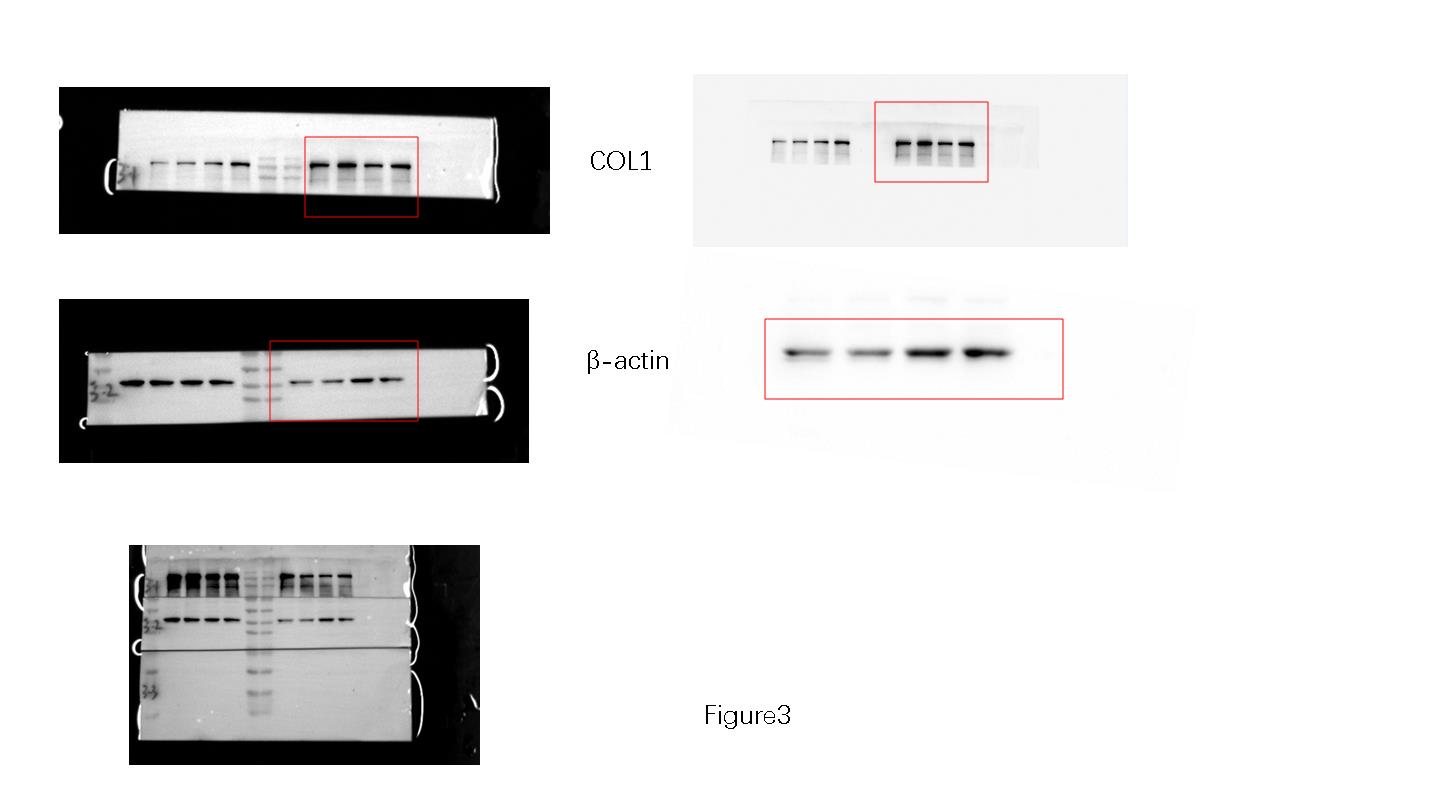

Supplement: Supplementary file 1 [file Data_Sheet_1.zip › 幻灯片4.jpeg]

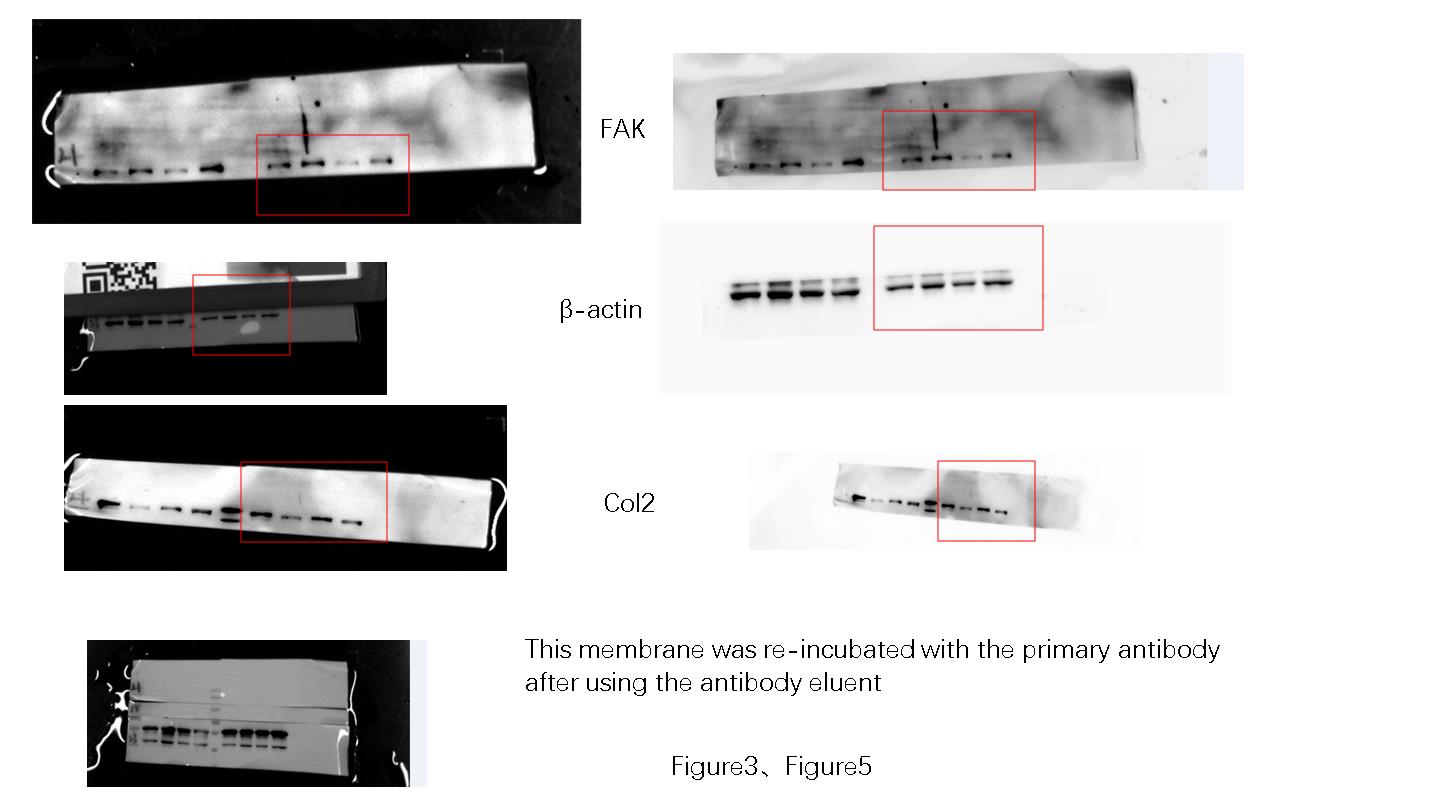

Supplement: Supplementary file 1 [file Data_Sheet_1.zip › 幻灯片5.jpeg]

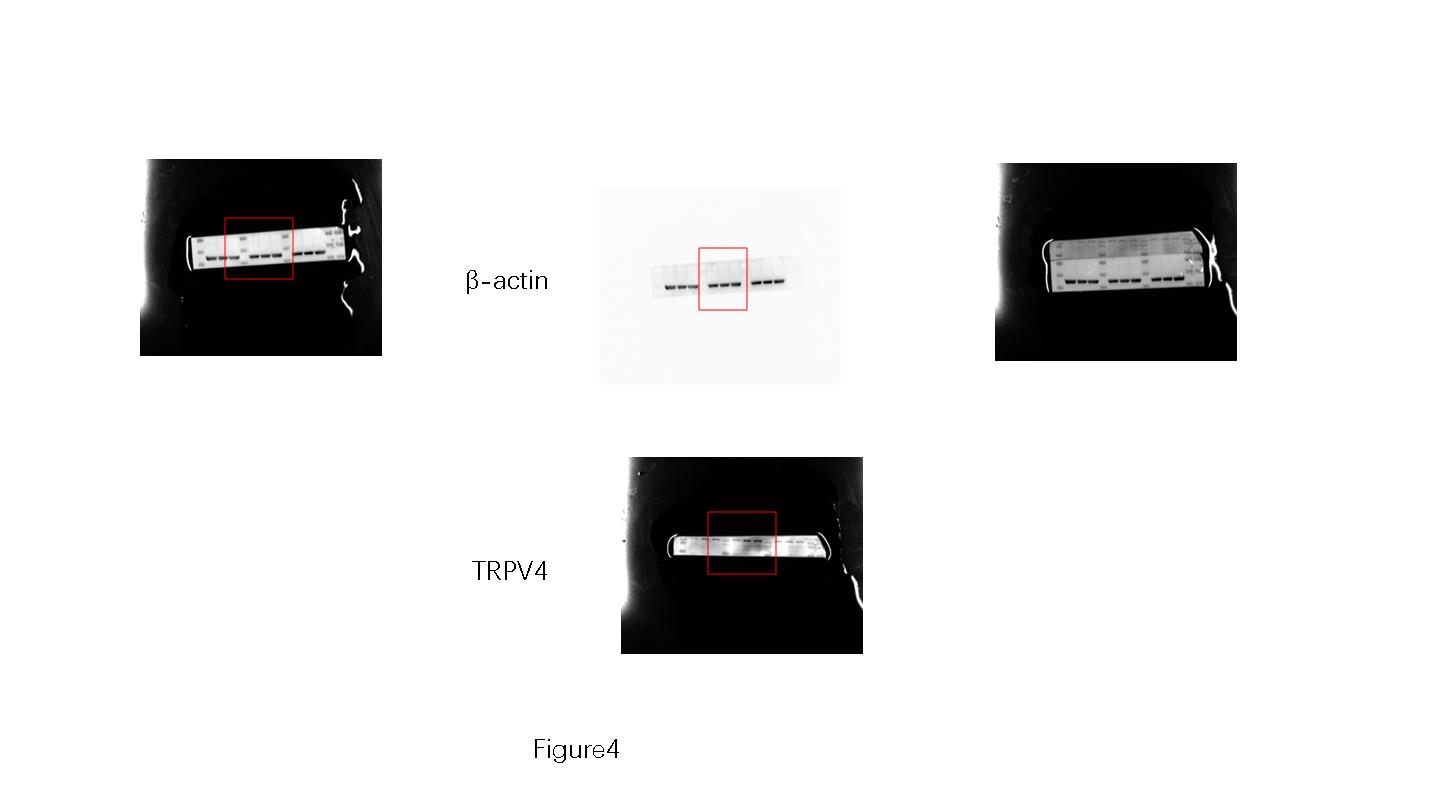

Supplement: Supplementary file 1 [file Data_Sheet_1.zip › 幻灯片6.jpeg]

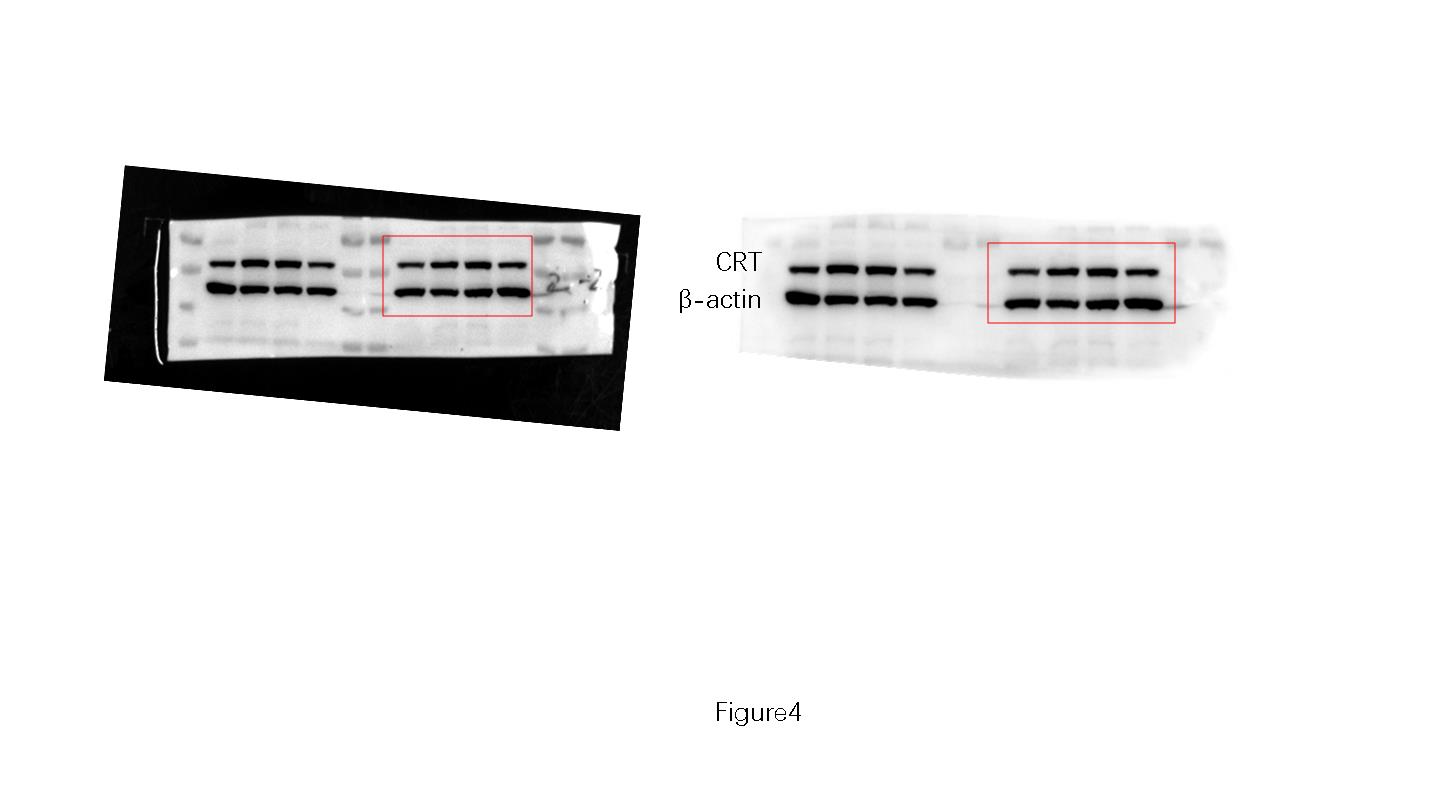

Supplement: Supplementary file 1 [file Data_Sheet_1.zip › 幻灯片7.jpeg]

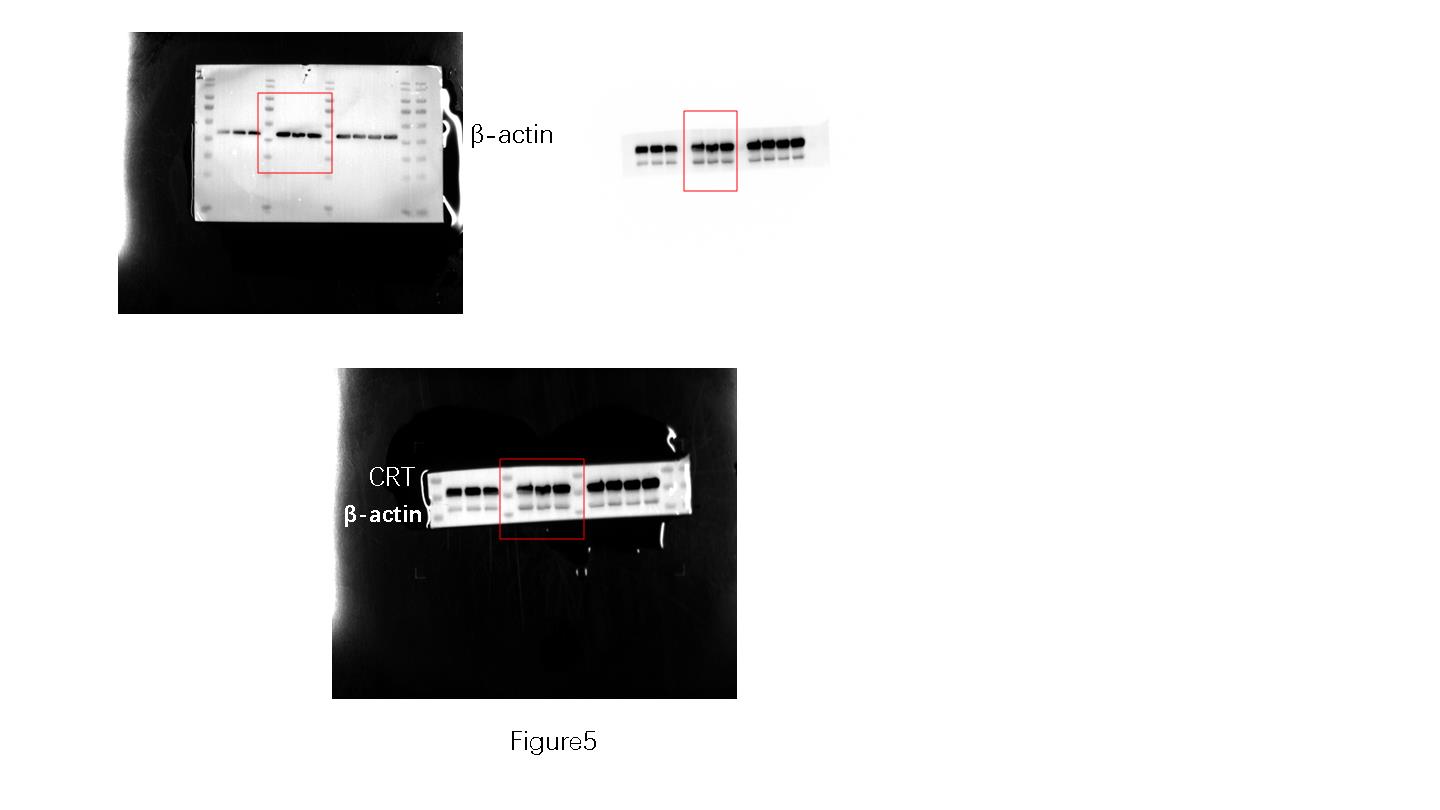

Supplement: Supplementary file 1 [file Data_Sheet_1.zip › 幻灯片8.jpeg]

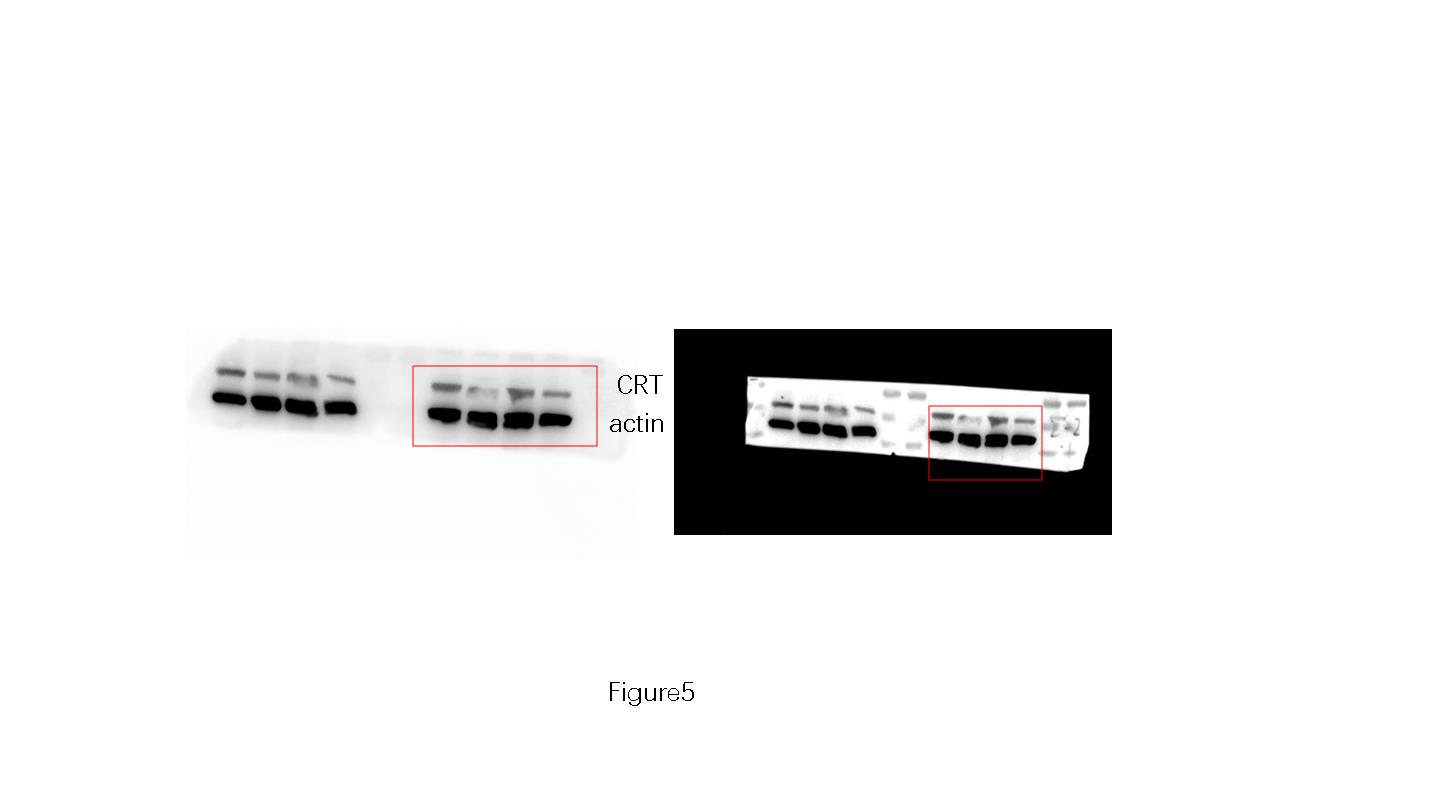

Supplement: Supplementary file 1 [file Data_Sheet_1.zip › 幻灯片9.jpeg]

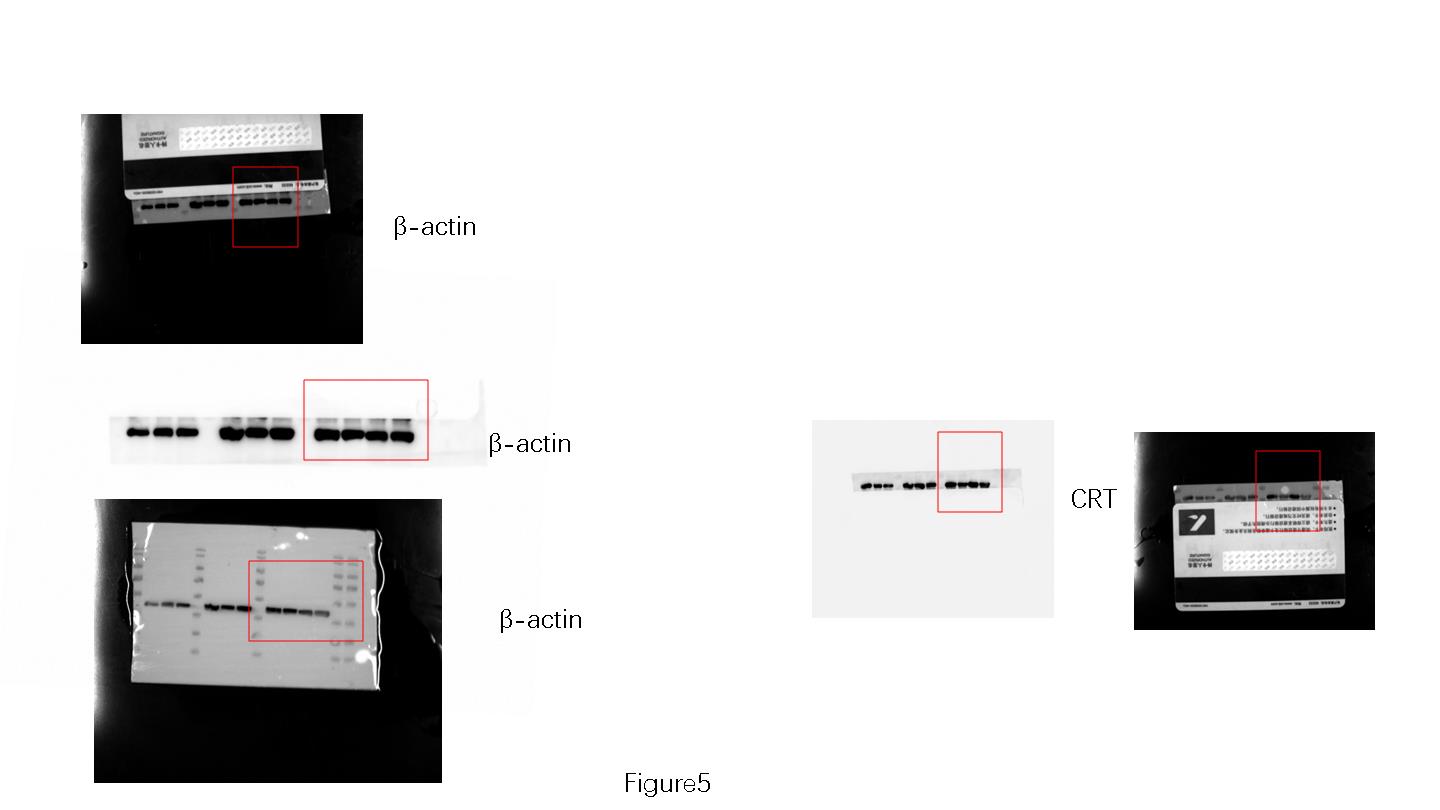

Supplement: Supplementary file 1 [file Data_Sheet_1.zip › 幻灯片10.jpeg]

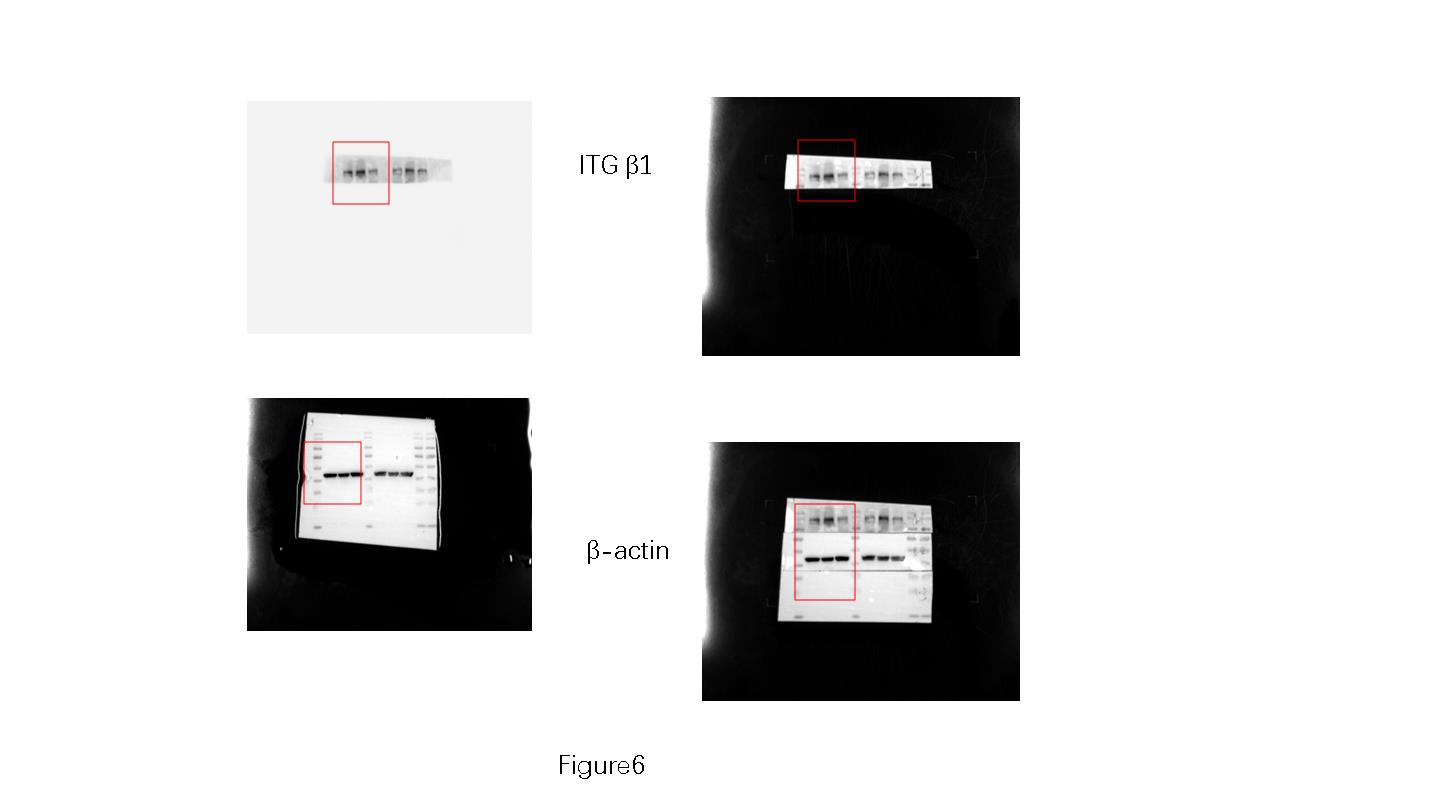

Supplement: Supplementary file 1 [file Data_Sheet_1.zip › 幻灯片11.jpeg]

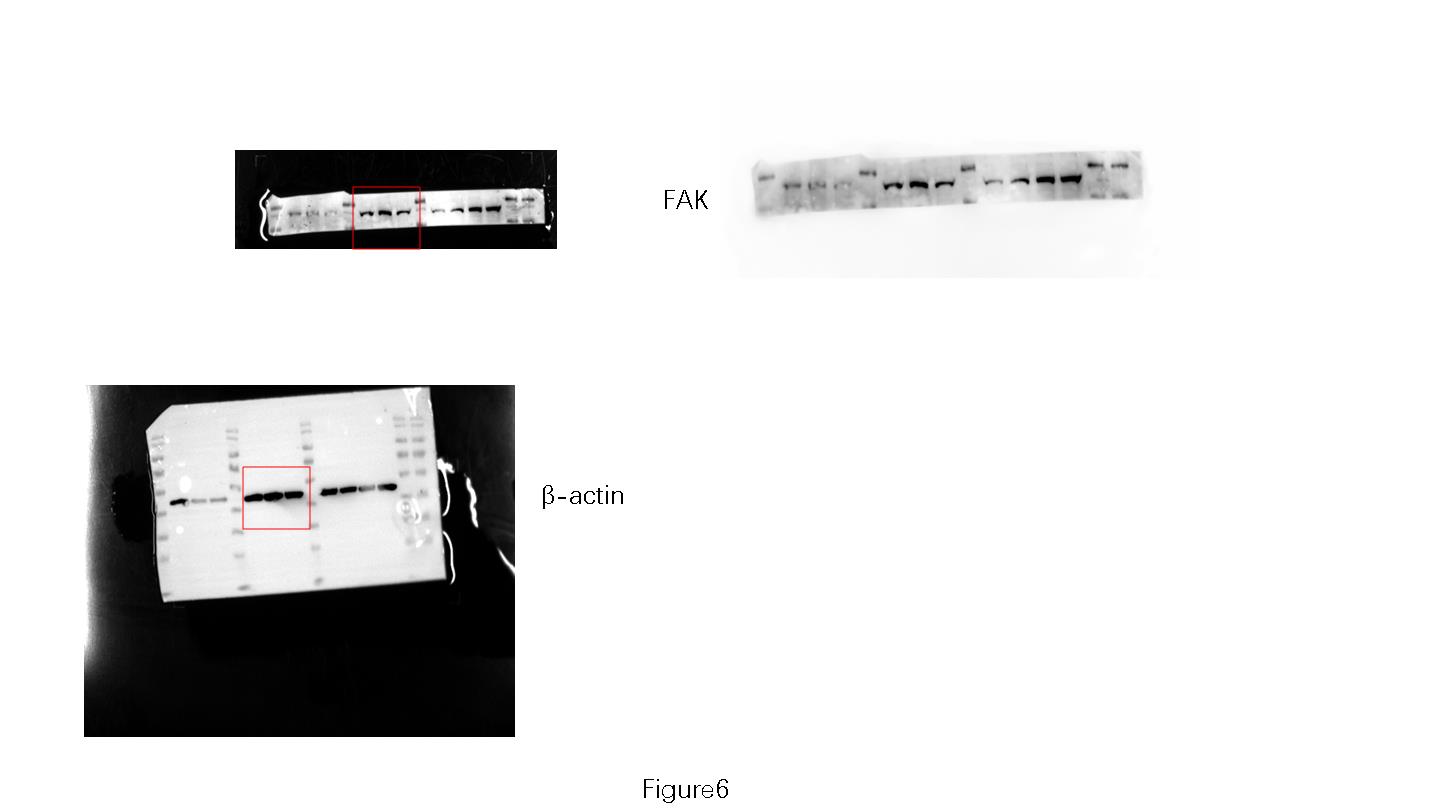

Supplement: Supplementary file 1 [file Data_Sheet_1.zip › 幻灯片12.jpeg]

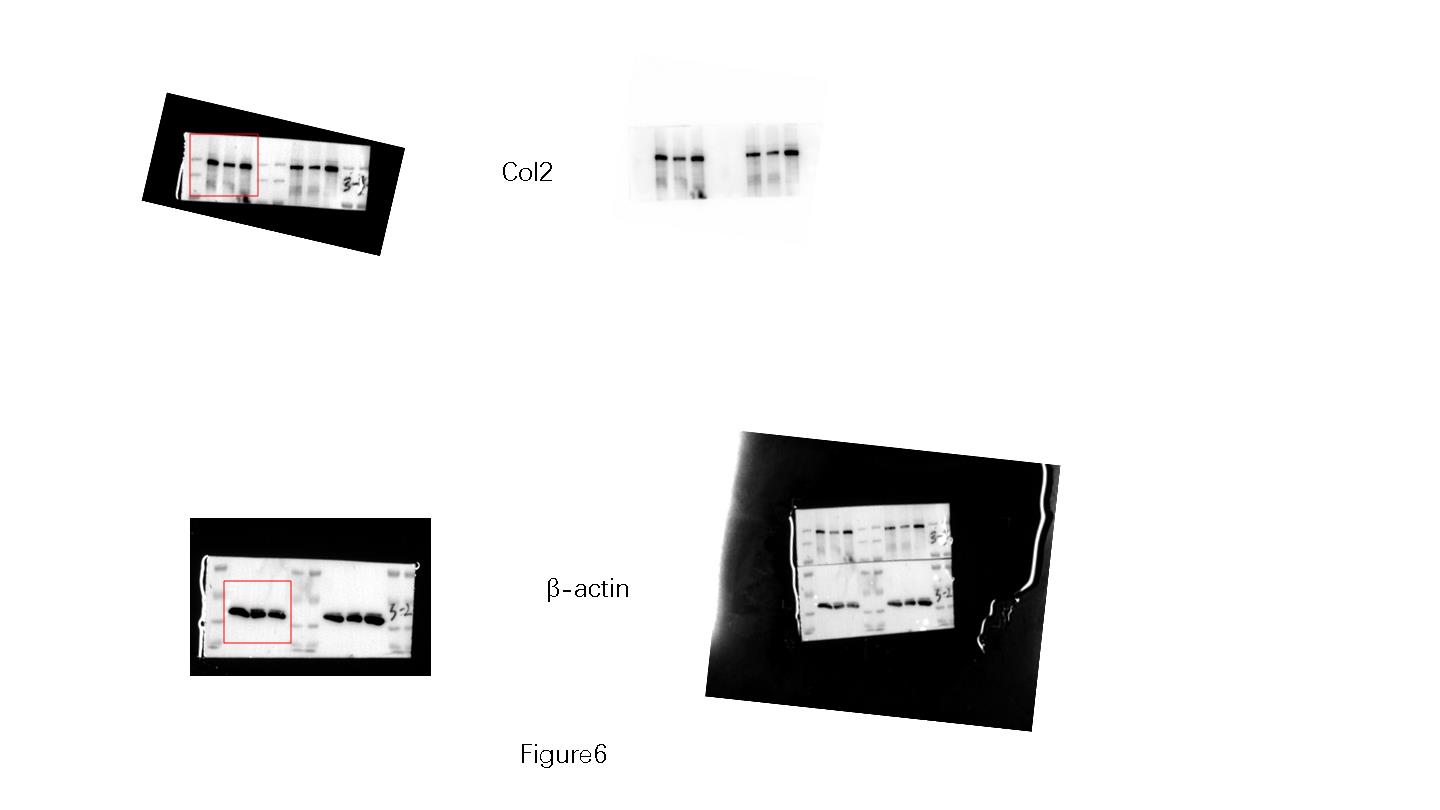

Supplement: Supplementary file 1 [file Data_Sheet_1.zip › 幻灯片13.jpeg]

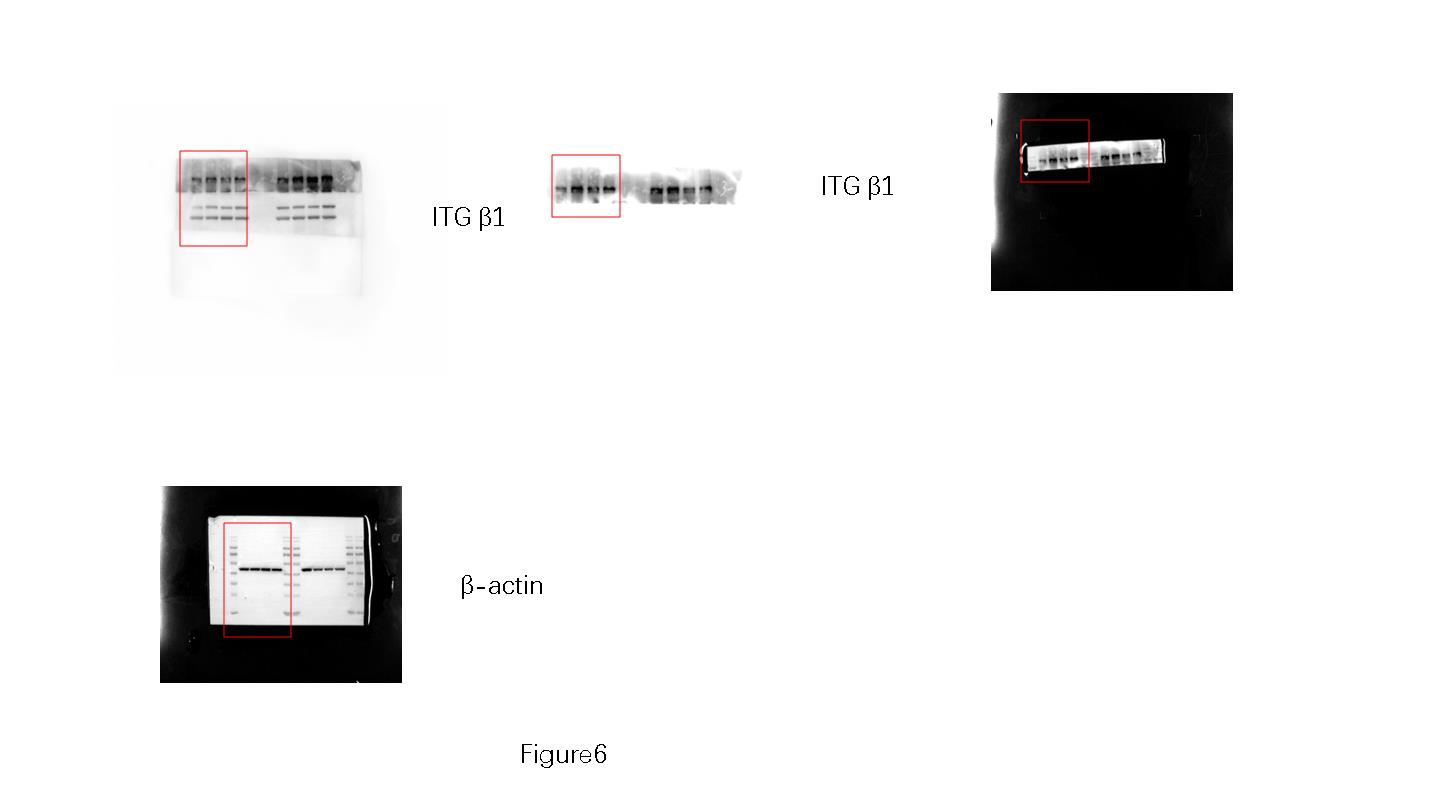

Supplement: Supplementary file 1 [file Data_Sheet_1.zip › 幻灯片14.jpeg]

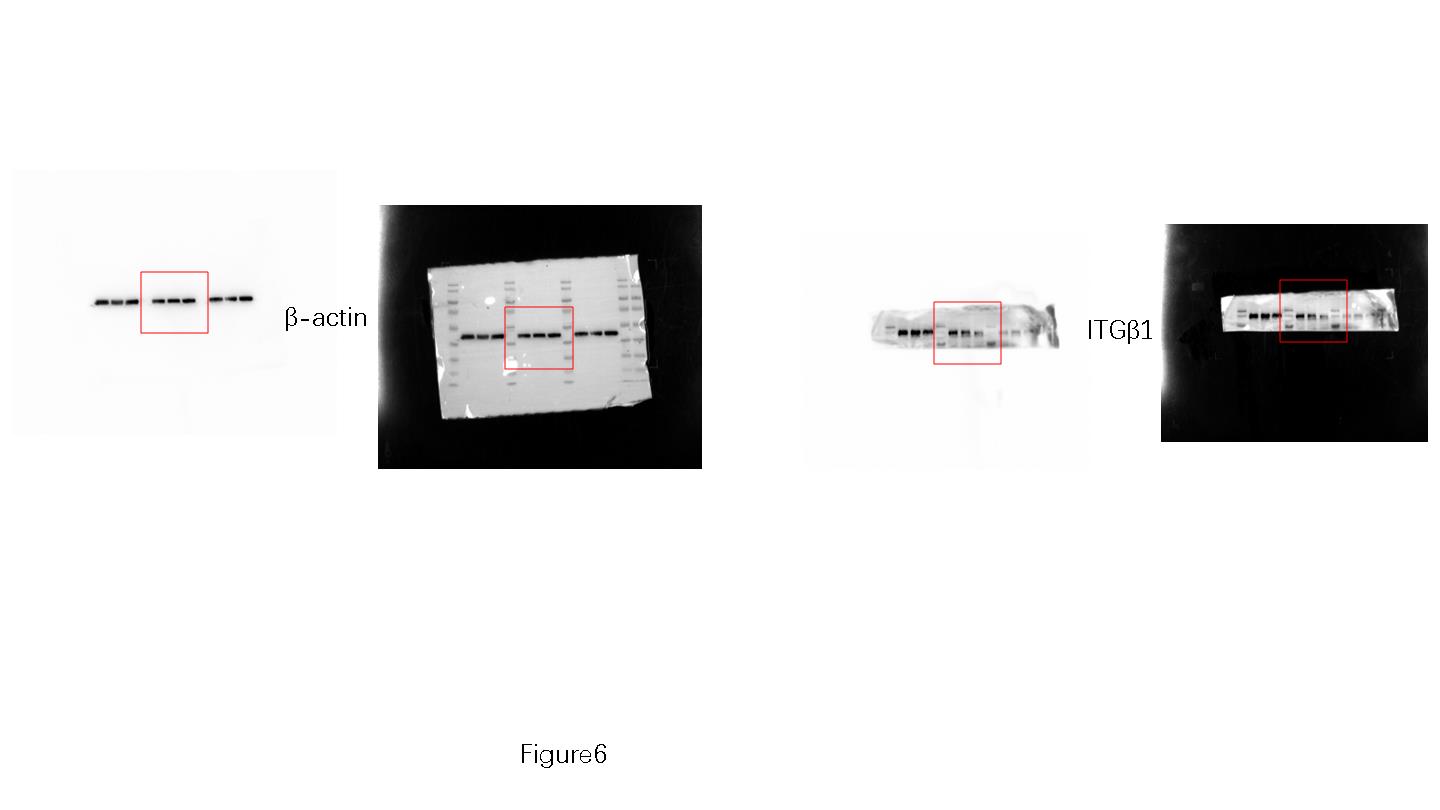

Supplement: Supplementary file 1 [file Data_Sheet_1.zip › 幻灯片15.jpeg]

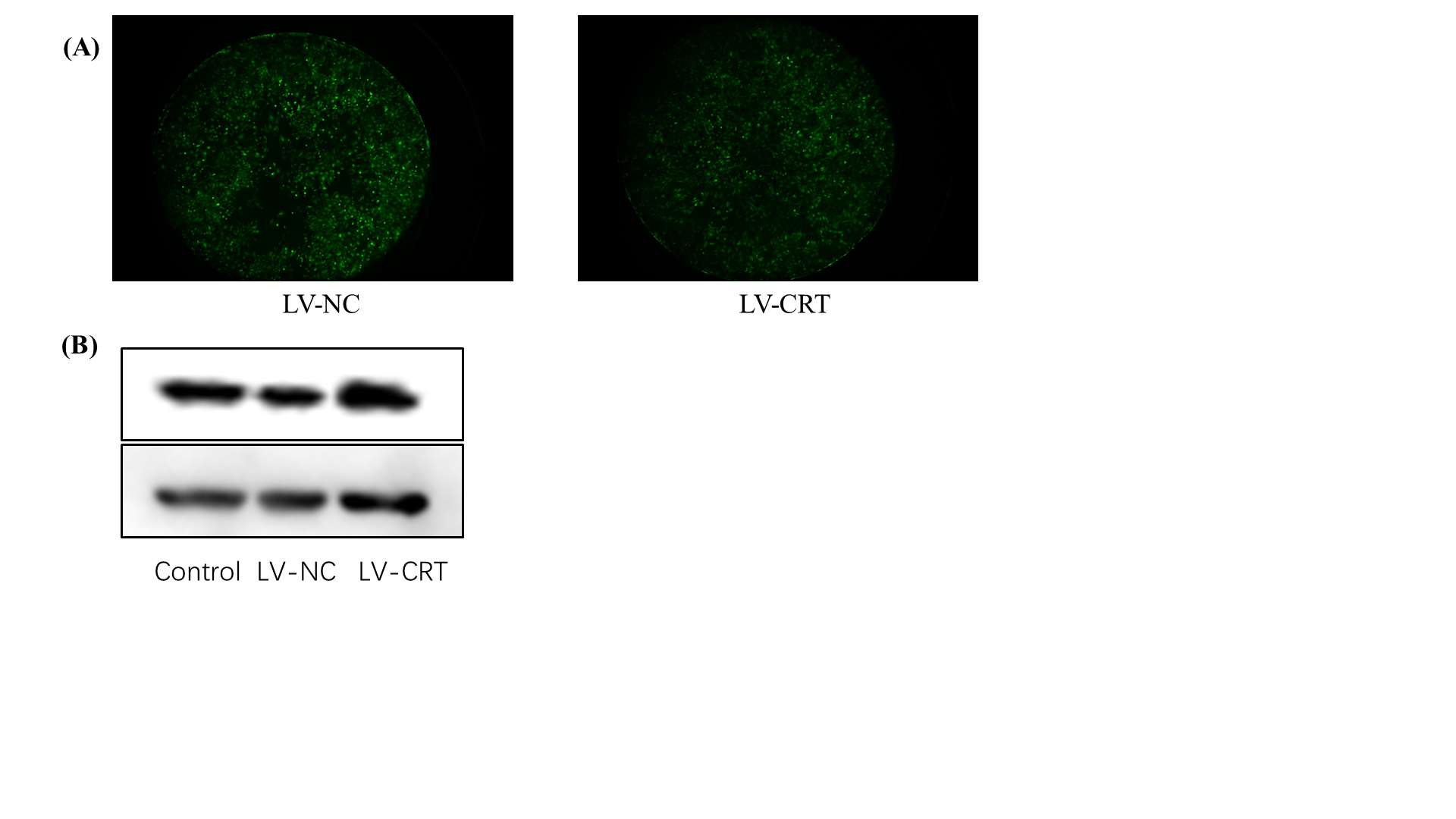

Supplement: Supplementary Figure S1 — CRT transfection. (A) The transfection efficiency was observed by immunofluorescence. (B) Western blot was used to detect protein expression after CRT transfection. [file Image_1.tiff]
